# Response to the Letter Entitled “Addressing the Limitations in Mobile Health Application Research for Oral Cancer Knowledge”

**DOI:** 10.1002/hsr2.70606

**Published:** 2025-03-24

**Authors:** Kehinde Kazeem Kanmodi, Afeez Abolarinwa Salami

**Affiliations:** ^1^ Faculty of Dentistry University of Puthisastra Phnom Penh Cambodia; ^2^ Department of Preventive and Community Dentistry University of Rwanda Kigali Rwanda; ^3^ Campaign for Head and Neck Cancer Education (CHANCE) Programme, Cephas Health Research Initiative Inc. Ibadan Nigeria; ^4^ School of Health and Life Sciences Teesside University Middlesbrough UK; ^5^ School of Public Health University of Port Harcourt Port Harcourt Nigeria; ^6^ Department of Oral and Maxillofacial Surgery University College Hospital Ibadan Nigeria; ^7^ Department of Public Health Dentistry Manipal Academy of Higher Education Manipal India

Dear Editor,

We read the letter entitled “Addressing the Limitations in Mobile Health Application Research for Oral Cancer Knowledge” with great enthusiasm. The letter provided constructive feedback and further insights on the future directions concerning our mixed methods systematic review which was entitled “The Types and Effectiveness of Mobile Health Applications Used in Improving Oral Cancer Knowledge: A Mixed Methods Systematic Review” [1].

In summary, the letter identified that we should (i) provide a more nuanced depiction of the effectiveness of the two mobile health applications—M‐OncoED and Prayaas—we [2, 3, 4] reviewed, in terms of their knowledge, clinical, behavioral, and epidemiological outcomes; (ii) provide further clarity on the offline functionality and compatibility of M‐OncoED—one of the reviewed educational mobile health applications—on basic mobile phones and the implications of the limitations of the application's usability in low‐resource settings; (iii) address the limitations of our review and emphasize the need for a more robust research on the use of educational mobile health applications where a more geographically diverse population groups are involved; and (iv) recommend the need for a very robust randomized control trial to test the effectiveness of the applications we reviewed.

The above‐summarized recommendations (i–iv) are very insightful and responding to these recommendations is worthwhile. In addressing recommendation “i” (see above), we would like to first provide a nuanced depictions of the effectiveness of the reviewed applications using a table (Table 1). With the use of a table, we believe that the similarity of contrasts between the two reviewed applications are now presented in a more simplified and nuanced way.

**Table 1:** The attributes and the level of significance of the effectiveness of the reviewed mobile health applications (M‐OncoED and Prayaas).

Application attributes and its effectiveness across tested domains	M‐OncoED	Prayaas	References
**Attributes**			
Target users	Physicians	Patients, lay persons, and healthcare providers	[3, 4]
Educational content	Content was focused on oral cancer, breast cancer, and cervical cancer	Content was focused on oral cancer only	[3, 4]
Tested Domains*			
Level of significance of change in knowledge outcomes after use	Not significant (*p* > 0.05)	Significant (*p* < 0.0001)	[2, 3, 4]
Level of significance of change in behavioral outcomes after use	Significant (*p* < 0.05) (the application, after use, enhanced physicians' behaviors toward patient counseling on oral cancer screening)	No data was reported on this	[2, 3, 4]
Level of significance of change in clinical outcomes after use	Not significant (*p* > 0.05) (the application, after use, did not enhance oral cancer screening practices among physicians)	No data was reported on this	[2, 3, 4]
Level of significance of change in epidemiological outcomes after use	No data was reported on this	No data was reported on this	[2, 3, 4]

*Note: p* = *p*‐value; *A *p*‐value below 0.05 was used to determine the level of statistical significance in the reviewed articles, i.e., a *p*‐value below 0.05 was considered to be statistically significant while a *p*‐value equal to or above 0.05 was considered to be statistically insignificant.

In response to recommendation “ii,” we would like to mention that the inability of M‐OncoED to work offline makes it unusable in locations where there is poor (or lack of) access to internet facility, making the application not equitable enough for public use. Additionally, locations with poor (or lack of) access to internet are predominantly low‐resource settings which are characterized with high burden of oral diseases, including oral cancer. To ensure an equitable access to oral cancer education interventions delivered via mobile health applications, it becomes imperative that educational mobile health applications that can dually work offline and online are created for public use.

In response to recommendations “iii” and “iv,” we would like to recommend the creation of a more inclusive and comprehensive educational mobile health application on oral cancer through the use of participatory action research methodologies, which can thereafter be tested via a systematically organized randomized control trial conducted among a geographically diverse lay population.

In addition to the above responses to recommendations “iii” and “iv,” we would like to note that we are very enthusiastic about conducting future empirical research studies that will address the gaps identified in our systematic review [1]. In January 2025, we published a study protocol on how we will cocreate a more comprehensive and inclusive educational mobile health application prototype on oral cancer and, thereafter, test the application prototype among first‐year university students across four countries: India, Nigeria, Rwanda, and Sri Lanka [5]. Once we have tested the created application prototype, we hope to launch it as a full‐fledged application and roll it out for public use.

In conclusion, our receipt of the letter entitled “Addressing the Limitations in Mobile Health Application Research for Oral Cancer Knowledge” clearly indicates that the use of mobile health applications in educating the public on oral cancer is garnering attention in the academic research space; this is a reflection of high hopes that oral cancer education delivered through the use of mobile health applications will be increasingly accessible to diverse populations across the world [6].

## Author Contributions

**Kehinde Kazeem Kanmodi:** conceptualization, investigation, funding acquisition, writing – original draft, methodology, validation, visualization, writing – review and editing, software, formal analysis, project administration, data curation, supervision, resources. **Afeez Abolarinwa Salami:** writing – review and editing.

## Ethics Statement

This study did not collect data from human or animal subjects but from an open research repository.

## Conflicts of Interest

Kehinde Kazeem Kanmodi is an Editorial Board member of Health Science Reports and a co‐author of this article. To minimize bias, they were excluded from all editorial decision‐making related to the acceptance of this article for publication. Other authors have no conflicts of interest to declare.

## Transparency Statement

The manuscript guarantor, Kehinde Kazeem Kanmodi, affirms that this manuscript is an honest, accurate, and transparent account of the study being reported; that no important aspects of the study have been omitted; and that any discrepancies from the study as planned (and, if relevant, registered) have been explained.

## Data Availability

The authors have nothing to report. All authors have read and approved the final version of the manuscript. Kehinde Kazeem Kanmodi, who is the corresponding author and manuscript guarantor, had full access to all of the data in this study and take complete responsibility for the integrity of the data and the accuracy of the data analysis.
